# Inactivation of staphylococcal virulence factors using a light-activated antimicrobial agent

**DOI:** 10.1186/1471-2180-9-211

**Published:** 2009-10-05

**Authors:** Sarah Tubby, Michael Wilson, Sean P Nair

**Affiliations:** 1 Division of Microbial Diseases, UCL Eastman Dental Institute, 256 Gray's Inn Road, WC1X 8LD, UK

## Abstract

**Background:**

One of the limitations of antibiotic therapy is that even after successful killing of the infecting microorganism, virulence factors may still be present and cause significant damage to the host. Light-activated antimicrobials show potential for the treatment of topical infections; therefore if these agents can also inactivate microbial virulence factors, this would represent an advantage over conventional antibiotic therapy. *Staphylococcus aureus *produces a wide range of virulence factors that contribute to its success as a pathogen by facilitating colonisation and destruction of host tissues.

**Results:**

In this study, the ability of the light-activated antimicrobial agent methylene blue in combination with laser light of 665 nm to inactivate staphylococcal virulence factors was assessed. A number of proteinaceous virulence factors were exposed to laser light in the presence of methylene blue and their biological activities re-determined. The activities of V8 protease, α-haemolysin and sphingomyelinase were shown to be inhibited in a dose-dependent manner by exposure to laser light in the presence of methylene blue.

**Conclusion:**

These results suggest that photodynamic therapy could reduce the harmful impact of preformed virulence factors on the host.

## Background

*Staphylococcus aureus *is a versatile opportunistic pathogen that causes a variety of infections ranging from superficial skin infections to severe, invasive diseases such as bacteraemia and necrotising pneumonia [1]. Its success as a human pathogen is highlighted by the fact that despite the development of antibiotic therapy, the frequency of staphylococcal bacteraemias is increasing [2]. Developments in medicine can at least be partly attributed to this rise as an increasing number of staphylococcal infections are related to the use of joint prostheses, immunosuppressants and catheters [3]. Indeed, *S. aureus *is the most frequent cause of surgical site infections, accounting for 38% of infections reported in the UK during the period January 2003 to December 2007 [4].

Methicillin-resistant *S. aureus *(MRSA) accounts for a high proportion of surgical site infections caused by *S. aureus*, being responsible for 64% of such infections in 2007/2008 [4]. Fewer than 5% of *S. aureus *isolates are now sensitive to penicillin, once the drug of choice for staphylococcal infections [5]. MRSA was first reported in the United Kingdom just two years after the introduction of methicillin in 1959 [6]. Horizontal transfer of the *mecA *gene, which encodes a penicillin-binding protein, results in resistance not only to methicillin, but also to broad spectrum β-lactams such as the third-generation cephalosporins, cefamycins and carbapenems [7]. The proportion of MRSA isolates from blood cultures taken from cases of bacteraemia in England has risen dramatically from less than 5% in 1990 to around 40% by the end of the 1990s [4]. As well as mortality rates of almost double those associated with methicillin-sensitive *S. aureus *(MSSA) infections, MRSA has put a considerable financial burden on both hospitals and society in general [8].

Over 40 different virulence factors have been identified in *S. aureus*; these are involved in almost all processes from colonisation of the host to nutrition and dissemination [9]. *S. aureus *produces a wide range of enzymes and toxins that are thought to be involved in the conversion of host tissues into nutrients for bacterial growth [10] in addition to having numerous modulatory effects on the host immune response [11].

The increasing resistance of pathogenic bacteria such as *S. aureus *to antibiotics has led to the search for new antimicrobial strategies, and photodynamic therapy (PDT) is emerging as a promising alternative. The photodynamic inactivation of bacteria relies upon the capacity of a light-activated antimicrobial agent (or "photosensitiser") to generate reactive oxygen species on irradiation with light of a suitable wavelength. Reactive oxygen species can oxidise many biological structures such as proteins, nucleic acids and lipids. As the mechanism of action of microbial killing is non-specific and multiple sites are affected, it is considered unlikely that resistance will evolve [12], thus representing a significant advantage over conventional antibiotic treatment where resistance is an ever-increasing problem. A very desirable feature of PDT is the potential for inactivation of virulence factors, particularly secreted proteins, by reactive oxygen species [13].

The biological activities of some virulence factors produced by Gram-negative bacteria have been shown to be successfully reduced by photodynamic action. The inhibitory effect of red laser light and the photosensitiser Toluidine Blue O (TBO) on virulence factors from *Escherichia coli, Pseudomonas aeruginosa *[14] and *Porphyromonas gingivalis *[15] has previously been demonstrated; however the effect on staphylococcal virulence factors has not been investigated.

In this study, we have investigated the effect of photosensitisation using methylene blue and laser light of 665 nm on some of the key virulence factors of *S. aureus*. The use of methylene blue is well established in medicine where it is used for the routine staining of vital organs and the treatment of septic shock [16].

## Results

### EMRSA-16

**Figure 1:**
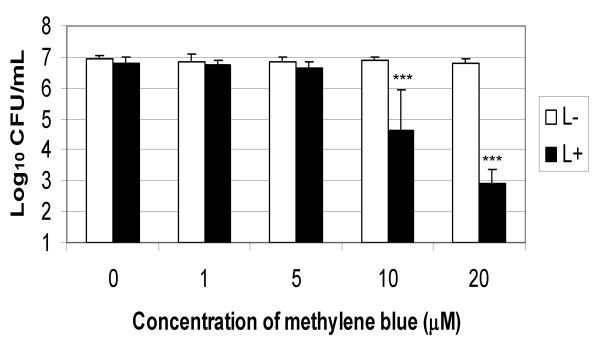
**Lethal photosensitisation of EMRSA-16 with 1, 5, 10 and 20 μM methylene blue and a 665 nm laser light dose of 1.93 J/cm^2^**. An equal volume of either PBS (S-) or methylene blue (S+) (concentrations ranging from 1-20 μM) was added to 50 μL of the bacterial suspension and either kept in the dark (L-) (white bars) or exposed to 665 nm laser light with an energy density of 1.93 J/cm^2 ^(L+) (black bars). After irradiation/dark incubation, samples were serially diluted and the surviving CFU/mL enumerated. Error bars represent the standard deviation from the mean. *** *P *< 0.001 (Mann Whitney *U *test). Experiments were performed three times in triplicate and the combined data are shown.

**Figure 2:**
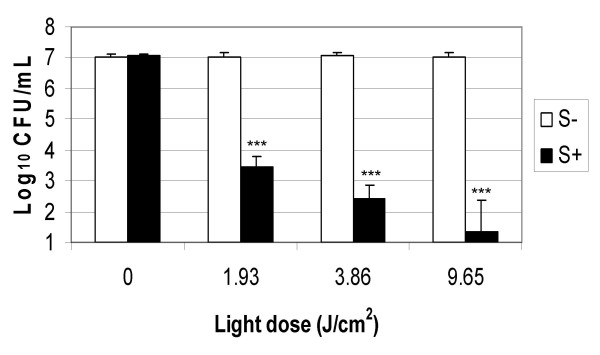
**The effect of 20 μM methylene blue and laser light doses of 1.93 J/cm^2^, 3.86 J/cm^2 ^and 9.65 J/cm^2 ^on the lethal photosensitisation of EMRSA-16**. An equal volume of either PBS (S-) (white bars) or 20 μM methylene blue (S+) (black bars) was added to 50 μL of the bacterial suspension and either kept in the dark (L-) or exposed to 665 nm laser light for 1, 2 and 5 minutes, corresponding to energy densities of 1.93 J/cm^2^, 3.86 J/cm^2 ^and 9.65 J/cm^2 ^(L+). After irradiation/dark incubation, samples were serially diluted and the surviving CFU/mL enumerated. Error bars represent the standard deviation from the mean. *** *P *< 0.001 (Mann Whitney *U *test). Experiments were performed three times in triplicate and the combined data are shown.

Methylene blue and laser light of 665 nm was found to successfully kill EMRSA-16, as shown by Figures 1 and 2. Treatment of EMRSA-16 with 20 μM methylene blue and a laser light dose of 1.93 J/cm^2 ^resulted in an approximate 4-log reduction in viability, corresponding to 99.98% kill. After irradiation with 9.65 J/cm^2 ^laser light in the presence of 20 μM methylene blue, an approximate 6-log reduction in viability was achieved, corresponding to a 99.999% kill, demonstrating the effectiveness of this regimen against MRSA.

### V8 protease

**Figure 3:**
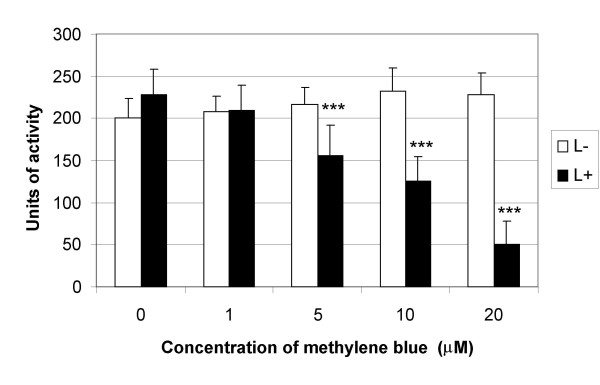
**The effect of methylene blue dose and 1.93 J/cm^2 ^laser light on the proteolytic activity of V8 protease**. An equal volume of either methylene blue (S+) (concentrations ranging from 1-20 μM) or PBS (S-) was added to V8 protease and samples were either exposed to laser light with an energy density of 1.93 J/cm^2 ^(L+) (black bars) or kept in the dark (L-) (white bars). The activity of the V8 protease was assessed using the azocasein hydrolysis assay. Error bars represent the standard deviation from the mean. *** *P *< 0.001 (ANOVA). Experiments were performed three times in triplicate and the combined data are shown.

**Figure 4:**
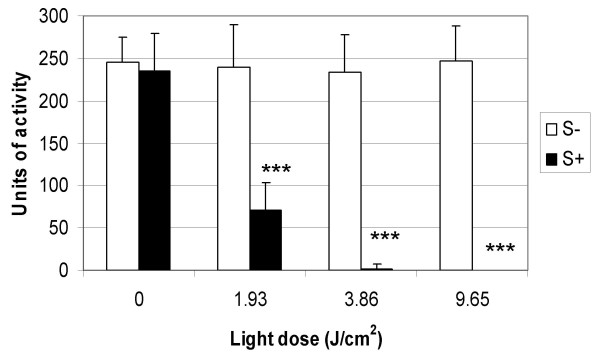
**The effect of 20 μM methylene blue and different laser light doses on the proteolytic activity of V8 protease**. V8 protease was either kept in the dark (L-) or irradiated with laser light doses of 1.93 J/cm^2^, 3.86 J/cm^2 ^and 9.65 J/cm^2 ^(L+) in the presence of an equal volume of either PBS (S-) (white bars) or 20 μM methylene blue (S+) (black bars). Following irradiation, the activity of the enzyme was assessed using the azocasein hydrolysis assay. Error bars represent the standard deviation from the mean. *** *P *< 0.001 (ANOVA). Experiments were performed three times in triplicate and the combined data are shown.

**Figure 5:**
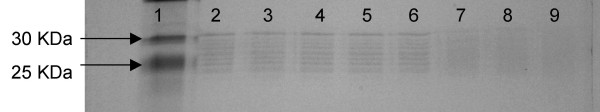
**SDS PAGE analysis of V8 protease irradiated with methylene blue and laser light doses of 1.93 J/cm^2^, 3.86 J/cm^2 ^and 9.65 J/cm^2^**. V8 protease was either kept in the dark (L-) or irradiated with laser light doses of 1.93 J/cm^2^, 3.86 J/cm^2 ^and 9.65 J/cm^2 ^(L+) in the presence of an equal volume of either PBS (S-) or 20 μM methylene blue (S+). Following irradiation, samples were analysed by SDS PAGE using a 5% stacking gel and 15% resolving gel under denaturing conditions. Lane 1: molecular weight marker, lane 2: L-S-, lane 3: L-S+, lane 4: L+S- (1.93 J/cm^2^), lane 5: L+S- (3.86 J/cm^2^), lane 6: L+S- (9.65 J/cm^2^), lane 7: L+S+ (1.93 J/cm^2^), lane 8: L+S+ (3.86 J/cm^2^), lane 9: L+S+ (9.65 J/cm^2^). L = samples exposed to laser light and S = samples exposed to 20 μM methylene blue. The apparent molecular mass of the V8 protease was approximately 30 kDa.

The effect of methylene blue and laser light on the proteolytic activity of the V8 protease as determined by the azocasein-hydrolysis assay is shown in Figures 3 and 4. One unit of activity was defined as that which caused a change in absorbance of 0.001 in one hour at 450 nm. Figure 3 shows that the activity of the V8 protease was inhibited in a photosensitiser concentration-dependent manner, with a decrease in proteolytic activity of 75% being achieved with the highest concentration of methylene blue tested (20 μM) upon irradiation for 1 minute with laser light (1.93 J/cm^2^). Photosensitisation of EMRSA-16 using the same conditions resulted in an approximate 4-log reduction in viability, showing that inactivation of this enzyme is effective within the parameters required to kill *S. aureus in vitro*. Figure 4 shows the effect of light dose on the activity of the V8 protease after exposure to laser light for 1, 2 and 5 minutes, corresponding to energy densities of 1.93 J/cm^2^, 3.86 J/cm^2 ^and 9.65 J/cm^2 ^respectively. Inactivation was also seen to be light dose-dependent and a 100% reduction in proteolytic activity was achieved following 5 minutes irradiation with laser light in the presence of 20 μM methylene blue. Neither laser light nor methylene blue alone had an inhibitory effect on the activity of the V8 protease. SDS PAGE analysis (Figure 5) showed that after exposure to laser light and methylene blue, the bands derived from the V8 protease appeared to be progressively more smeared and of lower intensity with increased irradiation time, demonstrating that photosensitisation may cause a change in the protein, perhaps due to oxidation of the protein. A band of 29 kDa was expected for the V8 protease; however the gel showed some degradation of the V8 protease that could not be inhibited by the addition of a protease inhibitor.

### α-haemolysin

**Figure 6:**
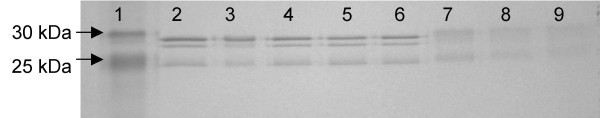
**SDS PAGE analysis of α-haemolysin irradiated with 20 μM methylene blue and laser light doses of 1.93 J/cm^2^, 3.86 J/cm^2 ^and 9.65 J/cm^2^**. α-haemolysin was either kept in the dark (L-) or irradiated with laser light doses of 1.93 J/cm^2^, 3.86 J/cm^2 ^and 9.65 J/cm^2 ^(L+) in the presence of an equal volume of either PBS (S-) or 20 μM methylene blue (S+) Following irradiation, samples were analysed by SDS PAGE using a 5% stacking gel and 15% resolving gel under denaturing conditions. Lane 1: molecular weight marker, lane 2: L-S-, lane 3: L-S+, lane 4: L+S- (1.93 J/cm^2^), lane 5: L+S- (3.86 J/cm^2^), lane 6: L+S- (9.65 J/cm^2^), lane 7: L+S+ (1.93 J/cm^2^), lane 8: L+S+ (3.86 J/cm^2^), lane 9: L+S+ (9.65 J/cm^2^). L = samples exposed to laser light and S = samples exposed to 20 μM methylene blue. The apparent molecular mass of α-haemolysin was approximately 29 kDa.

Table 1 shows the effect of photosensitisation of α-haemolysin with 1, 5, 10 and 20 μM methylene blue and laser light. Concentrations of 5, 10 and 20 μM methylene blue completely inhibited the haemolytic activity of the enzyme when exposed to laser light (L+); therefore inactivation of the toxin occurs even at photosensitiser doses that are sub-inhibitory to EMRSA-16 (i.e. 5 μM). There was no effect on the activity when the enzyme was incubated with the methylene blue in the absence of laser light (L-). To investigate the effect of light dose on the activity of α-haemolysin, the enzyme was exposed to 20 μM methylene blue and irradiated with 665 nm laser light for 1, 2 and 5 minutes. Table 2 shows that the activity of the enzyme was completely inhibited after exposure to a light dose of 1.93 J/cm^2 ^in the presence of 20 μM methylene blue, and further investigation showed that a laser light dose as low as 0.64 J/cm^2 ^results in the complete inhibition of haemolytic activity when treated with 20 μM methylene blue (data not shown). Laser light alone had no appreciable effect on the activity of the α-haemolysin. SDS PAGE analysis (Figure 6) showed that bands derived from the α-haemolysin after photosensitisation with 20 μM methylene blue and laser light became less well defined and smeared with increasing irradiation time compared to untreated samples. This result is similar to that observed for the V8 protease. The addition of 12.5% human serum did not affect the ability of photosensitisation to inactivate the α-haemolysin, and complete inhibition of haemolytic activity was observed after treatment of the toxin with 20 μM methylene blue and a laser light dose of 1.93 J/cm^2 ^in the presence of serum. This finding is consistent with the inactivation of the toxin in the absence of serum.

**Table 1:** The effect of treatment of α-haemolysin with different concentrations of methylene blue and a laser light dose of 1.93 J/cm^2^.

Concentration of methylene blue (μM)	Haemolytic titre L-	Haemolytic titre L+
1	1/1024	1/256

5	1/1024	1/2

10	1/1024	< 1/2

20	1/512	1/2

An equal volume of either 1, 5, 10 and 20 μM methylene blue or PBS was added to *S. aureus *α-haemolysin and samples were either exposed to 1.93 J/cm^2 ^laser light (L+) or kept in the dark (L-). After irradiation/dark incubation, samples were serially diluted and an equal volume of 4% rabbit erythrocytes was added. Following incubation in the dark at 37°C for one hour, the haemolytic titre was recorded. The haemolytic titre is defined as the highest dilution giving rise to haemolysis. Experiments were performed twice in duplicate and a representative experiment is shown.

**Table 2:** The effect of light dose on the activity of α-haemolysin when treated with 20 μM methylene blue

Light Dose (J/cm^2^)	Haemolytic titre S-	Haemolytic titre S+
0	1/512	1/512

1.93	1/256	< 1/2

3.86	1/256	< 1/2

9.65	1/256	< 1/2

An equal volume of either 20 μM methylene blue (S+) or PBS (S-) was added to *S. aureus *α-haemolysin and samples were either exposed to laser light doses of 1.93 J/cm^2^, 3.86 J/cm^2 ^and 9.65 J/cm^2 ^(L+) or kept in the dark (L). After irradiation/dark incubation, samples were serially diluted and an equal volume of 4% rabbit erythrocytes was added. Following incubation in the dark at 37°C for one hour, the haemolytic titre was recorded. The haemolytic titre is the highest dilution giving rise to haemolysis. Experiments were performed twice in triplicate and a representative experiment is shown.

### Sphingomyelinase

**Figure 7:**
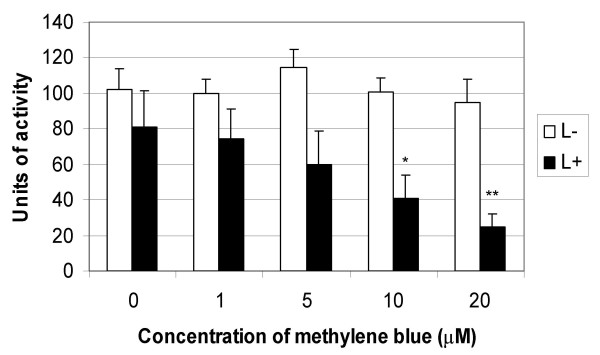
**The effect of methylene blue dose when irradiated with 1.93 J/cm^2 ^laser light on the activity of *S. aureus *sphingomyelinase**. An equal volume of either 1, 5, 10 and 20 μM methylene blue (S+) or PBS (S-) was added to sphingomyelinaseand samples were either exposed to laser light with an energy density of 1.93 J/cm^2 ^(L+) (black bars) or kept in the dark (L-) (white bars). Following irradiation, the activity of the enzyme was assessed spectrophotometrically using the substrate TNPAL-Sphingomyelin. Error bars represent the standard deviation from the mean. *** *P *< 0.001 (ANOVA). Experiments were performed three times in duplicate and the combined data are shown.

**Figure 8:**
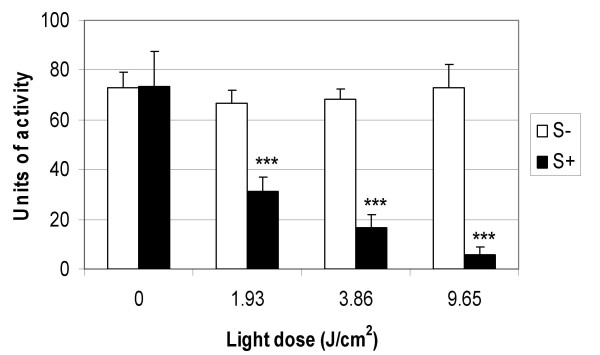
**The effect of 20 μM methylene blue when irradiated with different laser light doses on the activity of *S. aureus *sphingomyelinase**. Sphingomyelinase was either kept in the dark (L-) or irradiated with laser light doses of 1.93 J/cm^2^, 3.86 J/cm^2 ^and 9.65 J/cm^2 ^(L+) in the presence of an equal volume of either PBS (S-) (white bars) or 20 μM methylene blue (S+) (black bars). Following irradiation with laser light doses of 1.93 J/cm^2^, 3.86 J/cm^2 ^and 9.65 J/cm^2^, the activity of the enzyme was assessed spectrophotometrically using the substrate TNPAL-Sphingomyelin. Error bars represent the standard deviation from the mean. *** *P *< 0.001 (ANOVA). Experiments were performed three times in triplicate and the combined data are shown.

The activity of *S. aureus *sphingomyelinase was inhibited by treatment with methylene blue and laser light in a dose-dependent manner, as shown in Figures 7 and 8. One unit of activity was defined as that which caused a change in absorbance of 0.001 in one minute at 330 nm. Interestingly, laser light alone appeared to have a slight effect on the activity of the enzyme, although this was not statistically significant (*P *> 0.05). Irradiation with 1.93 J/cm^2 ^laser light in the presence of 20 μM methylene blue achieved a 76% decrease in the activity of sphingomyelinase, which is comparable to the decrease in activity seen for the V8 protease (75%); these photosensitisation conditions correspond to an approximate 4-log reduction in viable EMRSA-16 and therefore inactivation is effective with light and energy doses required for the effective killing of bacteria. After irradiation with 9.65 J/cm^2 ^laser light in the presence of 20 μM methylene blue, a decrease in activity of 92% was observed. Neither dark incubation of the enzyme with the photosensitiser alone nor irradiation in the absence of methylene blue had a significant effect on the activity of the sphingomyelinase. Irradiation of the sphingomyelinase in the presence of 12.5% human serum did not have an effect on the ability of photosensitisation to inactivate the enzyme. Photosensitisation using 20 μM methylene blue and the lowest laser light dose (1.93 J/cm^2^) resulted in a decrease in the enzyme's activity of 70% ± 12% in the presence of human serum, compared to a decrease of 76% ± 10% in the absence of serum. This difference was not found to be statistically significant (*P *> 0.05, ANOVA).

## Discussion

Packer *et al*. [15] demonstrated that proteolytic enzymes of the periodontal pathogen *Porphyromonas gingivalis *could be inactivated using the photosensitiser Toluidine Blue O and red laser light with a wavelength of 633 nm. The results presented here support these findings, with a highly significant reduction in the activity of *S. aureus *V8 protease being achieved with a light dose as low as 1.93 J/cm^2 ^in combination with a low (20 μM) concentration of methylene blue. This inactivation was found to be dose-dependent, with the highest concentration of methylene blue tested (20 μM) and irradiation with 9.65 J/cm^2 ^laser light achieving a 100% reduction in activity compared to non-treated samples. Treatment of EMRSA-16 under the same conditions resulted in a 99.999% kill, indicating that inactivation of secreted proteases may be possible as well as eradicating infecting bacteria.

Proteases are responsible for the destruction of host tissues both directly and indirectly; inactivation of these enzymes using PDT could therefore limit the damage to the host, as well as killing the infecting organism. It has been suggested that theV8 protease plays an important role in the pathogenesis of *S. aureus*, as strains lacking this enzyme show reduced virulence in a number of infection models [17-19]. Of particular relevance is a murine abscess model, in which inactivation of V8 protease resulted in significant attenuation of virulence [18]; therefore inactivation of this enzyme by PDT may be able to reduce the virulence potential of *S. aureus *in other hosts.

As staphylococcal proteases and the colonisation of atopic skin by *S. aureus *have been implicated in the pathogenicity of atopic dermatitis [20], the inactivation of proteolytic enzymes could have particular relevance for the decontamination of infected lesions using PDT. PDT using the combination of methylene blue and laser light of 665 nm may therefore be of use in the treatment of atopic skin disorders. In fact, PDT has long been shown to be beneficial in the treatment of atopic skin disorders, for example the use of ultraviolet light and 8-methoxypsoralen for the treatment of atopic dermatitis [21]. Clearly, the combination of elimination of disease-exacerbating microorganisms and neutralisation of virulence factors would be extremely advantageous to the treatment of these diseases.

The treatment of α-haemolysin with methylene blue and laser light resulted in an effective inhibition of haemolytic activity. Concentrations of methylene blue ranging from 1-20 μM all had an inhibitory effect on α-haemolysin when irradiated with laser light, and α-haemolysin was shown to be inactivated after an irradiation time of just 1 minute (1.93 J/cm^2^) when in the presence of 20 μM methylene blue. The results shown here demonstrate that α-haemolysin is the most susceptible of the virulence factors tested, perhaps due to the nature of its amino acid composition, which may leave it more vulnerable to attack by reactive oxygen species. These data indicate that photodynamic inactivation of this toxin is highly effective and as such, could significantly attenuate the virulence of *S. aureus *due to the multiple functions of α-haemolysin as a virulence factor.

The haemolysins of *S. aureus *are membrane-damaging toxins that are capable of lysing a number of different cell types. α-haemolysin is thought to be important in infection as it has a number of detrimental effects on host cells due to the disruption of ion transport across host cell membranes, ultimately leading to apoptotic cell death and oedema [10]. α-haemolysin can cause cell death in different ways depending on the concentration of the toxin. At high concentrations, α-haemolysin forms large pores in lipid bilayers that result in massive necrosis, whilst low doses result in the formation of small pores that result in apoptosis and DNA fragmentation [22]. The role of α-haemolysin in the virulence of *S. aureus *has been demonstrated in a number of infection models such as mastitis [23] and pneumonia [24]. It has also been proposed that α-haemolysin may play a role in colonisation of epithelia by attenuating bacterial clearance from the epithelial surface [25]; this could therefore be of relevance to the decontamination of nasal epithelia using PDT. In addition, α-haemolysin has immunomodulatory properties, notably its ability to trigger the release of pro-inflammatory cytokines such as interleukin-1β [26]; thus inactivation of α-haemolysin by PDT may also protect against harmful inflammatory processes as well as eliminating infecting organisms.

The treatment of *S. aureus *sphingomyelinase with laser light and methylene blue resulted in a significant, dose-dependent reduction in the enzyme's activity. Laser light alone also appeared to reduce the activity of sphingomyelinase; however this was found to be not statistically significant. Irradiation of sphingomyelinase with 1.93 J/cm^2 ^laser light in the presence of the highest concentration of methylene blue tested (20 μM) achieved a highly significant reduction in the activity of the enzyme (76%), which was comparable to the reduction in activity observed for the V8 protease when irradiated for the same time period. This reduction in activity was increased to 92% after irradiation of the enzyme for 5 minutes in the presence of 20 μM methylene blue. Production of sphingomyelinase (β-haemolysin) is thought to be of importance in severe, chronic skin infections, and strains of *S. aureus *producing high levels of this enzyme have been shown to cause more intense skin lesions than low-producing strains [27]. Inactivation of these toxins may therefore be of notable relevance to the treatment of superficial staphylococcal skin infections. Sphingomyelinase has recently been shown to kill proliferating T lymphocytes, suggesting a role for this toxin in evasion of the host immune response [28]; hence inactivation of sphingomyelinase by PDT could also reduce the immunomodulatory properties of *S. aureus*.

The photodynamic inactivation of α-haemolysin and sphingomyelinase was shown to be unaffected by the presence of human serum at concentrations resembling the protein content of an acute wound[29], indicating that photodynamic therapy may be effective in inactivating these virulence factors *in vivo*. Together with the data showing that PDT using methylene blue and 665 nm laser light is effective against a methicillin-resistant strain of *S. aureus*, this supports the potential of PDT as a treatment for superficial staphylococcal infections.

The precise mechanism of inhibition of these virulence factors has not yet been determined; however it is possible that the reactive oxygen species formed during photosensitisation can oxidise proteins, thereby disrupting their function [13]. Oxidation of active site groups has been suggested as the mechanism of action for the photodynamic inactivation of the proteolytic enzymes of *P. gingivalis *[15]. SDS PAGE analysis of the V8 protease and α-haemolysin demonstrated that photosensitisation caused changes to the proteins which resulted in smearing of the protein bands. We propose that singlet oxygen may play a role in the inactivation of V8 protease as a protective effect is observed when photosensitisation is performed in the presence of the singlet oxygen scavenger L-tryptophan (data not shown).

## Conclusion

In conclusion, the results of this study suggest that photosensitisation with methylene blue and laser light of 665 nm may be able to reduce the virulence potential of *S. aureus*, as well as effectively killing the organism. Inactivation of α-haemolysin and sphingomyelinase is not affected by the presence of human serum, indicating that PDT may be effective against these toxins *in vivo*. Considering the extensive damage virulence factors can cause to host tissues, the ability to inhibit their activity would be a highly desirable feature for any antimicrobial treatment regimen and would represent a significant advantage over conventional antibiotic strategies.

## Methods

### Light source

A Periowave™ laser (Ondine Biopharma Inc., Canada), which emits light with a wavelength of 665 nm was used for all irradiation experiments. For experimental purposes, the laser system was set up to give a power density of 32 mW/cm^2^. The power output of the laser was measured using a thermopile power meter (TPM-300CE, Genetic, Canada) and was found to be 73 mW at the plate surface.

### Photosensitiser

Methylene blue (C_16_H_18_ClN_3_S.3H_2_O) was purchased from Sigma-Aldrich (UK). Stock solutions of 0.1 mg/ml were prepared in phosphate buffered saline (PBS) and kept in the dark at room temperature.

### Bacterial strains

EMRSA-16 was maintained by weekly subculture on Blood Agar (Oxoid Ltd, UK), supplemented with 5% horse blood (E & O Laboratories Ltd). For experimental purposes, bacteria were grown aerobically in Brain Heart Infusion broth (Oxoid Ltd, UK) at 37°C for 16 hours in a shaking incubator at 200 rpm. Cultures were centrifuged and resuspended in an equal volume of PBS and the optical density was adjusted to 0.05 at 600 nm, corresponding to approximately 1 × 10^7 ^colony forming units (CFU) per mL.

### The effect of photosensitiser dose on the lethal photosensitisation of EMRSA-16

Methylene blue was diluted in PBS to give final concentrations of 1, 5, 10 and 20 μM. 50 μL of methylene blue was added to an equal volume of the inoculum in triplicate wells of a sterile, flat-bottomed, untreated 96-well plate and irradiated with 665 nm laser light with an energy density of 1.93 J/cm^2 ^(L+S+), with stirring. Three additional wells containing 50 μL methylene blue and 50 μL of the bacterial suspension were kept in the dark to assess the toxicity of the photosensitiser alone (L-S+). 50 μL PBS was also added to 50 μL of the inoculum in a further six wells, three of which were irradiated with laser light as above (L+S-) and the remaining three were kept in the dark (L-S-). Following irradiation/dark incubation, each sample was serially diluted 10-fold in PBS. 10 μL of each dilution was spotted onto 5% horse blood agar plates in triplicate and the plates incubated aerobically overnight at 37°C. The surviving CFU/mL were enumerated by viable counting. Experiments were performed three times in triplicate.

### The effect of laser light dose on the lethal photosensitisation of EMRSA-16

Methylene blue was diluted in PBS to give a final concentration of 20 μM. 50 μL of methylene blue was added to an equal volume of the inoculum in triplicate wells of a sterile, flat-bottomed, untreated 96-well plate and irradiated with 665 nm laser light with energy densities of 1.93 J/cm^2^, 3.86 J/cm^2 ^or 9.65 J/cm^2^, corresponding to 1, 2 or 5 minutes irradiation respectively, with stirring (L+S+). Three additional wells containing 50 μL methylene blue and 50 μL of the bacterial suspension were kept in the dark (L-S+) and 50 μL PBS was also added to 50 μL of the inoculum in a further six wells, three of which were irradiated with laser light (L+S-) and the remaining three were kept in the dark (L-S-). Following irradiation/dark incubation, each sample was serially diluted 10-fold in PBS. 10 μL of each dilution was spotted onto 5% horse blood agar plates in triplicate and the plates incubated aerobically overnight at 37°C. The surviving CFU/mL were enumerated by viable counting. Experiments were performed three times in triplicate.

### Azocasein hydrolysis assay

Endoproteinase Glu-C (also known as V8 protease) from *S. aureus *V8 was purchased from Sigma-Aldrich (UK) and stored at -20°C at a concentration of 1 mg/mL in dH_2_O. A final concentration of 5 μg/mL was obtained by diluting the enzyme in PBS after preliminary experiments to determine the appropriate concentration for the assay conditions. 50 μL of V8 protease was added to an equal volume of either methylene blue (S+) or PBS (S-) in triplicate wells of a 96-well plate and samples were irradiated with laser light (L+) or incubated in the dark (L-). For photosensitiser dose experiments, final concentrations of 1, 5, 10 and 20 μM methylene blue were used and samples were irradiated with 665 nm laser light with an energy density of 1.93 J/cm^2^. For laser light dose experiments, a final concentration of 20 μM methylene blue was used and samples were irradiated with 665 nm laser light for either 1, 2 or 5 minutes, corresponding to energy densities of 1.93 J/cm^2^, 3.86 J/cm^2 ^or 9.65 J/cm^2^.

After irradiation, the azocasein hydrolysis assay (modified from [15]) was performed. 100 μL was removed from each well and added to 50 μL of 6% azocasein (w/v) in 0.5 M Tris buffer, pH 7 (Sigma-Aldrich, UK) in 0.5 mL Eppendorf tubes. Samples were incubated in the dark for one hour at 37°C. The reaction was stopped with an equal volume of 20% acetic acid and the samples centrifuged for 10 minutes at 5590 × *g*. 75 μL of the supernatant was removed in duplicate and the optical density read at 450 nm using a Dynex plate reader. The enzyme activity at one hour was calculated for each sample; one unit of activity was determined as that which caused a change in absorbance of 0.001 in one hour at 450 nm. Photosensitiser and light dose experiments were performed three times in triplicate.

### Haemolytic titration

α-haemolysin from *S. aureus *was purchased from Sigma-Aldrich (UK) and stored at 2-8°C at a concentration of 0.5 mg/mL in sterile, deionised water plus sodium citrate buffer. For experimental purposes, α-haemolysin was diluted in sterile PBS to a final concentration of 100 μg/mL after preliminary experiments to determine the appropriate concentration for the assay conditions and according to Bhakdi *et al*. [30]. For photosensitiser dose experiments, the stock solution of methylene blue was diluted in PBS to give final concentrations of 1, 5, 10 and 20 μM. 50 μL of methylene blue was added to an equal volume of α-haemolysin in duplicate wells of a sterile, flat-bottomed, untreated 96-well plate and irradiated with laser light for 1 minute, corresponding to an energy dose of 1.93 J/cm^2 ^(L+S+). Two additional wells containing 50 μL methylene blue and 50 μL of the α-haemolysin were kept in the dark to assess the effect of the photosensitiser alone (L-S+). 50 μL PBS was also added to 50 μL of the α-haemolysin in a further four wells, two of which were irradiated with laser light (L+S-) and the remaining two kept in the dark (L-S-). For laser light dose experiments, a final concentration of 20 μM methylene blue was used and samples were irradiated with 665 nm laser light for either 1, 2 or 5 minutes, corresponding to energy densities of 1.93 J/cm^2^, 3.86 J/cm^2 ^or 9.65 J/cm^2^. Following irradiation/dark incubation, samples were removed and aliquoted into round-bottomed 96-well plates for the haemolytic titration assay.

For the haemolytic titration assay, samples were serially diluted using doubling dilutions in PBS. Sterile, deionised water was used as a positive control and sterile PBS as a negative control. Defibrinated rabbit blood (E & O Laboratories, UK) was centrifuged at 503 × *g *for 10 minutes and the supernatant discarded. The cells were washed and resuspended in sterile PBS to a final concentration of 2%. 50 μL was added to the serially diluted toxin and control wells and incubated in the dark at 37°C for 1 hour. After incubation, the haemolytic titre for each sample was determined as the highest dilution giving rise to lysis. Photosensitiser dose experiments were performed twice in duplicate and light dose experiments were performed twice in triplicate

### The effect of human serum on the photosensitisation of *S. aureus *α-haemolysin

α-haemolysin was diluted to a final concentration of 100 μg/mL in either PBS or PBS + 12.5% human serum (Sigma Aldrich, UK) in order to determine the effect of serum on the photoinactivation of the toxin. 12.5% serum was added to model *in vivo *conditions as this concentration provided a protein concentration similar to that found in an acute wound [29]. α-haemolysin in either the presence or absence of human serum was exposed to 20 μM methylene blue and laser light with energy densities of 1.93 J/cm^2^, 3.86 J/cm^2 ^or 9.65 J/cm^2 ^and the haemolytic titration assay was performed as previously described. Experiments were performed twice in triplicate.

### Spectrophotometric assay for sphingomyelinase activity

Sphingomyelinase (also known as β-haemolysin or β-toxin) from *S. aureus *was purchased from Sigma-Aldrich (UK) in buffered aqueous glycerol containing 0.25 M phosphate buffer, pH 7.5. For experimental purposes, the enzyme was diluted to a final concentration of 0.5 Units/mL in 250 mM Tris-HCl buffer with 10 mM magnesium chloride, pH 7.4 at 37°C according to the manufacturer's instructions, based on the spectrophotometric assay for sphingomyelinase described by Gatt [31]. 25 μL of sphingomyelinase was added to either 25 μL of 1, 5, 10 or 20 μM methylene blue (S+) or 25 μL PBS (S-) and irradiation of the enzyme suspension was carried out using an energy density of 1.93 J/cm^2^, with the appropriate controls (L-S-, L-S+, L+S-). Experiments were performed three times in duplicate. For laser light dose experiments, 20 μM methylene blue and energy densities of 1.93 J/cm^2^, 3.86 J/cm^2 ^or 9.65 J/cm^2 ^were used and experiments were performed three times in triplicate

Following irradiation/dark incubation, the spectrophotometric assay for sphingomyelinase activity (modified from [32]) was performed. 10 μL from each sample was removed and added to 190 μL of incubation buffer containing 0.02 mg Trinitrophenylaminolauroyl-Sphingomyelin (TNPAL-Sphingomyelin; Sigma-Aldrich, UK), 250 mM Tris-HCl, 10 mM MgCl_2 _and 1% Triton X-100 in 0.5 mL Eppendorf tubes and incubated in the dark at 37°C for 5 minutes, with shaking. 150 μL of Isopropanol:Heptane:H_2_SO_4 _(40:10:1) was added to stop the reaction and the tubes were immediately placed on ice. 100 μL of n-heptane (Sigma-Aldrich, UK) and 80 μL deionised water were then added and the samples were centrifuged for ten minutes at 1398 × *g*. Following centrifugation, the tubes were left to settle at room temperature for 5 minutes, after which 60 μL of the upper layer was removed and the optical density at 330 nm recorded using a UV-VIS spectrophotometer. A blank sample containing 10 μL incubation buffer instead of sphingomyelinase was used as a reference.

### The effect of human serum on the photosensitisation of *S. aureus *sphingomyelinase

Sphingomyelinase was diluted to a final concentration of 0.5 Units/mL in either 250 mM Tris-HCl buffer with 10 mM magnesium chloride, pH 7.4 at 37°C or the buffer with the addition of 12.5% human serum (Sigma Aldrich, UK) in order to model acute wound conditions and exposed to 20 μM methylene blue and laser light with energy densities of 1.93 J/cm^2 ^or 9.65 J/cm^2^. The spectophotometric assay for sphingomyelinase activity was performed as previously described. Experiments were performed twice in triplicate.

### SDS PAGE analysis

After photosensitisation or dark incubation as previously described, the V8 protease and α-haemolysin were analysed by sodium dodecyl sulphate polyacrylamide gel electrophoresis (SDS PAGE). Briefly, 20 μL of each sample was added to 5 μL reducing SDS PAGE sample buffer (Pierce, UK) and boiled for 5 minutes to denature the protein. Samples were then analysed by SDS PAGE using a 5% stacking gel and 15% resolving gel. After electrophoresis, gels were placed in a fixative solution (40% methanol, 15% acetic acid) and then stained with Brilliant Blue G (Sigma, UK). V8 protease samples were incubated on ice with 100 mM phenylmethanesulfonyl fluoride for 30 minutes prior to SDS PAGE in order to minimise self-digestion. The expected molecular masses of the V8 protease and α-haemolysin were given as 29 kDa and 33 kDa respectively, as specified by the manufacturer.

### Statistical analysis

Data are expressed as means ± standard error. The results of the azocasein hydrolysis assay and sphingomyelinase assay were analysed using the univariate ANOVA test with Bonferroni analysis. The results from the lethal photosensitisation of EMRSA-16 were analysed using the Mann Whitney *U *test. For both statistical analyses, a *P *value of less than 0.05 was considered statistically significant. For photosensitiser dose experiments, the *P *values refer to samples in the absence of light versus irradiated samples. For light dose experiments, the *P *values refer to samples in the absence of methylene blue versus samples irradiated in the presence of methylene blue.

## Competing interests

Ondine Biopharma Inc. has funded and is continuing to fund this work. ST is receiving a student stipend from Ondine Biopharma Inc. for carrying out this work and MW holds shares in Ondine Biopharma Inc. Ondine Biopharma Inc. is also financing the article-processing charge.

## Authors' contributions

ST: participated in the study design, carried out the experimental work, performed the statistical analysis and drafted the manuscript. MW: conceived of the study, participated in its design and helped to draft the manuscript. SPN: conceived of the study, participated in its design, provided technical support and helped to draft the manuscript. All authors read and approved the final manuscript.
